# Serum calcitonin nadirs to undetectable levels within 1 month of curative surgery in medullary thyroid cancer

**DOI:** 10.20945/2359-3997000000112

**Published:** 2019-03-18

**Authors:** Fernanda Andrade, Geneviève Rondeau, Laura Boucai, Rebecca Zeuren, Ashok R. Shaha, Ian Ganly, Fernanda Vaisman, Rossana Corbo, Michael Tuttle

**Affiliations:** 1 Instituto Nacional de Câncer Instituto Nacional de Câncer Endocrinology Service Department of Medicine Rio de Janeiro RJ Brasil Department of Medicine, Endocrinology Service, Instituto Nacional de Câncer (INCA), Rio de Janeiro, RJ, Brasil; 2 Université de Montréal l’Université de Montréal Center Hospitalier Montreal Canadá Center Hospitalier de l’Université de Montréal, Medicine Endocrinology, Montreal, Canadá; 3 Memorial Sloan-Kettering Cancer Center Department of Medicine New York NY USA Department of Medicine, Endocrinology Service, Memorial Sloan-Kettering Cancer Center, New York, NY, USA; 4 Memorial Sloan-Kettering Cancer Center Department of Surgery, Head and Neck Cancer New York NY USA Department of Surgery, Head and Neck Cancer, Memorial Sloan-Kettering Cancer Center, New York, NY, USA

**Keywords:** Calcitonin, medullary thyroid cancer, risk stratification, curative surgery, prognostic

## Abstract

**Objective:**

Because serum calcitonin (CT) is a reliable marker of the presence, volume, and extent of disease in medullary thyroid cancer (MTC), both the ATA and NCCN guidelines use the 2-3 month post-operative CT value as the primary response to therapy variable that determines the type and intensity of follow up evaluations. We hypothesized that the calcitonin would nadir to undetectable levels within 1 month of a curative surgical procedure.

**Subjects and methods:**

This retrospective review identified 105 patients with hereditary and sporadic MTC who had at least two serial basal CT measurements done in the first three months after primary surgery.

**Results:**

When evaluated one year after initial surgery, 42 patients (42/105, 40%) achieved an undetectable basal calcitonin level without additional therapies and 56 patients (56/84, 67%) demonstrated a CEA within the normal reference range. In patients destined to have an undetectable CT as the best response to initial therapy, the calcitonin was undetectable by 1 month after surgery in 97% (41/42 patients). Similarly, in patients destined to have a normalize their CEA, the CEA was within the reference range by 1 month post-operatively in 63% and by 6 months in 98%. By 6 months after curative initial surgery, 100% of patients had achieved a nadir undetectable calcitonin, 98% had reached the CEA nadir, and 97% had achieved normalization of both the calcitonin and CEA.

**Conclusion:**

The 1 month CT value is a reliable marker of response to therapy that allows earlier risk stratification than the currently recommended 2-3 month CT measurement.

## INTRODUCTION

Medullary thyroid carcinoma (MTC) is a rare and challenging malignancy that often presents with loco-regional metastases and less commonly with distant metastases. Not infrequently these patients have persistent biochemical or structural evidence of disease after primary surgery that often displays a protracted indolent course (1–3). Prognosis largely depends on the presenting clinico-pathologic features as reflected by the American Joint Committee on Cancer (AJCC) staging (4), and on the completeness of surgical resection of the primary tumor and regional lymph nodes as assessed by the post-operative serum calcitonin (CT) (5–8).

Because serum CT is recognized to be a reliable biomarker of the presence, volume, and extent of disease in MTC (9–11), both the American Thyroid Association (ATA) and National Comprehensive Cancer Network (NCCN) guidelines post-operative CT and CEA values as the primary response to therapy variables that determines the type and intensity of follow up evaluations (2,3,8,12). While the ATA and NCCN guidelines recommend evaluating response to therapy at 2-3 months after initial therapy, several studies demonstrate that following curative surgery, serum calcitonin levels begin a rapid decline within hours after surgery (13) often achieving undetectable levels within the first few post-operative days (14–17). These data are consistent with our anecdotal clinical observations that surgically cured patients achieve undetectable calcitonin levels within 1 month of surgery even though the CEA values often required several months to normalize.

Therefore, the goal of this study was to determine if the 1 month post-operative CT value would identify patients that are destined to develop an undetectable calcitonin value following initial therapy. Rather than waiting two to three months to determine whether or not a curative surgery had been performed, we hypothesized that an undetectable serum calcitonin one month after surgery would identify the vast majority of patients destined to have an undetectable nadir calcitonin in response to initial therapy.

## SUBJECTS AND METHODS

### Subjects

After obtaining approval from the institutional review board, we retrospectively identified 105 MTC patients followed at Memorial Sloan Kettering Cancer Center in New York or the National Cancer Institute of Brazil in Rio de Janeiro (INCA) who had serial serum calcitonin measurements obtained using the same clinical assay at 1 month and 2-3 months after initial surgery. In patients that had both a 2 month and 3 month value, the value from month 3 was used for analysis. Thirty-nine patients also had a serum calcitonin in the same assay 6 months after primary surgery. Patients < 18 years old at diagnosis or those who did not have thyroid surgery as initial treatment for MTC were excluded from the study. The analysis of post operative calcitonin was performed in those patients with detectable calcitonin pre-operative.

### Calcitonin and CEA assays

The functional sensitivity of the calcitonin assays varied from 2 pg/mL in the Siemens DPC Immulite 2000-2500 Chemiluminescent Method (Quest Diagnostics, Madison, NJ, USA) to 8 pg/mL in the Siemens DPC Immulite ICMA Method (LabCorp, Burlington, NC, USA). Values below the functional sensitivity of the assay were classified as undetectable.

Serum CEA was measured using either the AIA-1800 IEMA Method (Tosoh, San Francisco, CA, USA) or the Siemens Chemiluminescent Method (Quest Diagnostics, Madison, NJ, USA). CEA values less than 5 ng/mL were considered to be within the expected reference range.

### Follow-up and Calcitonin measurements

In the first few months after initial surgery, basal CT measurements were ordered once a month for three months in order to obtain at least two serial values in the post-operative period. The next basal CT values were usually obtained at 6 and 12 months after primary surgery. Patients were seen in clinic every 3-6 months for the first year and at 6 month intervals thereafter at the discretion of the attending physician based on the risk of the individual patient and the clinical course of the disease. Since pentagastrin is not available in the United States (USA) or Brazil, no pentagastrin stimulated CT values were obtained in the patients included in the study.

### Statistical methods

Continuous data are presented as means and standard deviations or median values with ranges. Comparisons between groups were performed with one-way ANOVA test. Analysis was performed using SPSS software (Version 24.0.1: SPSS Inc, Chicago, IL). A p value ≤ 0.05 was considered statistically significant.

## RESULTS

The demographic and clinic-pathologic features of the 105 MTC patients analyzed in this study are presented in Table 1. More than half of the patients were female (53.3%) and median age at diagnosis was 53 years old. The majority were sporadic cases with a median duration of follow-up of 4.5 years.

**Table 1:** Clinical characteristics of entire cohort of 105 patients with medullary thyroid carcinoma

Age (years)	Median 53 Mean 52 ± 14 Range 8-84
Gender	Male 49 (46.7%) Female 56 (53.3%)
Follow up (years)	Median 4.5 Mean 4.8 ± 4.5 Range 0.5 – 23.4
Hereditary/Sporadic	Hereditary 15 (14.3%) Sporadic 78 (74.3%) Uncertain 12 (11.4%)
Extra thyroidal extension	Yes = 36 (34.3%) No = 69 (65.7%)
Vascular invasion	Yes = 45 (42.8%) No = 60 (57.2%)
AJCC staging	I = 33 (31.4%) II = 15 (14.3%) III = 6 (5.7%) IVa = 35 (33.4%) IVb = 1 (0.9%) IVc = 13 (12.4%) Not staged = 2 (1.9%)
Pre op CEA level (ng/mL) (n = 40)	Median = 32 Mean = 175 ± 57 Range = 0.5 – 1,708
Pre op CT levels (pg/mL) (n = 56)	Median = 1,435 Mean 25,998 ± 18,421 Range < 5 – 1,035,000

Considering the extent of the initial surgery, the vast majority was submitted to total thyrodectomy (102/105) and only 3 had hemithyrodectomy. Central lymph node dissection was perfomed in 64 patients and lateral compartment dissection in 56 patients. It is important to note that bilateral lymph node dissection were necessary in only 16 patients. From all the patients included in this study, 40% had cN1a disease and 44% cN1b. All 56 patients with lateral neck dissection were also submitted to central neck dissection as well. From the 64 patients with central neck dissection 26 had undetectable calcitonin. Some patients were operated because of RET mutation screening and some for clinically evident MTC, which had an impact on surgery choice. Due to the heterogeneous criteria for selecting the type of surgery performed, we were not able to show statistical significant correlation between the type of surgery and early biochemical remission.

When evaluated one year after initial surgery, 42 patients (42/105, 40%) achieved an undetectable basal calcitonin level without additional therapies (pre-operative CT ranged from 10 to 1,428 pg/mL, with the median value of 174 pg/mL). Furthermore, 56 patients (56/84, 67%) demonstrated a CEA within the normal reference range (21 patients did not have a CEA measured at the one year time point, pre-operative CEA ranged from 0.5 to 1,708 pg/mL with a median value of 174 pg/mL. Of the 84 patients with both calcitonin and CEA values available at the 1 year time point, 34 (34/84, 41%) achieved both an undetectable calcitonin and a CEA within the normal reference range.

**Figure 1:**
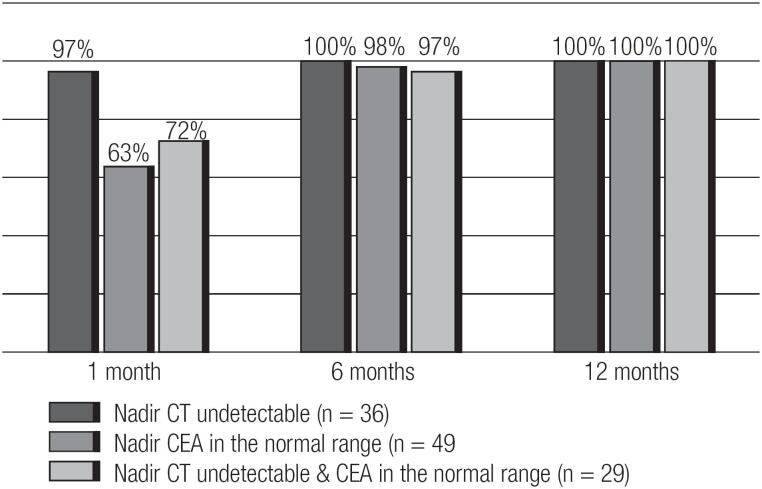
Time course to the normalization of the tumor makers in MTC patients surgically cured

The time course to achieving an (1) undetectable calcitonin, (2) normal range CEA and (3) both an undetectable calcitonin and CEA value is shown in Figure 1. Following initial curative surgery an undetectable calcitonin was achieved within the first month after surgery in 97% of the patients despite having pre-operative calcitonin values that ranged from 10 to 1,428 pg/mL. Interestingly, the 1 patient that failed to achieve an early undetectable calcitonin demonstrated a 1 month post-surgical calcitonin of only 5 pg/mL (assay limit of detection was 4 pg/mL). As expected, the CEA took longer to reach nadir with only 63% reaching the normal reference range by 1 month. However, by 6 months after curative initial surgery, 100% of patients had achieved a nadir calcitonin, 98% had reached the CEA nadir, and 97% had achieved normalization of both the calcitonin and CEA.

From the 42 patients that achieved undetectable calcitonin in the first year postoperative, 5 patients had detectable values at final follow up. However, only two cases had structural evidence of disease. Cases are described in Table 2.

**Table 2:** Patients with at least ine undetectable calcitonin whithin the first year that had elevated calcitonin at the end of follow-up

	Patient 1	Patient 2	Patient 3	Patient 4	Patient 5
Age	56	51	39	65	48
Sex	Male	Male	Female	Male	Female
Tumor size (cm)	1.0	2.5	2.5	1.0	2.6
N1	Yes	No	Yes	No	No
Type of initial surgery	Total thyroidectomy + central and lateral neck dissection	Hemithyroidectomy	Total thyroidectomy+ central neck dissection	Total thyroidectomy	Total thyroidectomy+ central neck dissection
Time to elevation of calcitonin after initial undetectable measurement	12 months	6 months	12 months	5 months	22 months
Last calcitonin	659	4.4	39	4.0	8.0
Structural disease at the end of follow up	Yes- distant metastases	No	Yes- small lymph nodes under survalliance	No	No
Final response to therapy stratification	Structural incomplete	Biochemical incomplete	Structural incomplete	Biochemical incomplete	Biochemical incomplete

## DISCUSSION

Consistent with previous observations, our data clearly show that following curative surgery, serum calcitonin levels rapidly decline and nearly always achieve an undetectable nadir within 1 month. While CEA levels may take longer to normalize, 63% had already achieved normal range values within 1 month of surgery. Thus our data demonstrate that measurements of calcitonin and CEA within the first month of surgery can provide an early, meaningful response to therapy assessment predictive of the likelihood that a surgical cure has been achieved. Conversely, very few patients will achieve an undetectable calcitonin and normal CEA levels more than 6 months after initial surgical intervention allowing the clinician and the patient to understand that they will likely have persistent biochemical and or structural evidence of disease as the best response to initial therapy.

Because of its reliability to reflect presence and extent of disease, there has been increased interest in determining how early after surgery CT levels would help predict the clinical course of the disease. Early series of patients demonstrated that 4/6 patients normalized their calcitonin 2 weeks after surgery (15) while 15/33 did so within three postoperative days (16). Ismailov and Piulatova reported CT normalization in 15/22 patients with MTC by 4 weeks after surgery and as early as 2 days postoperative (17). More recently, Bumming and cols. compared a CT value 6-8 weeks after surgery with a follow up calcitonin measured between 3 and 12 months. Five of seven patients with undetectable CT at 6-8 weeks continued to have undetectable levels at follow up. All 13/20 patients with detectable CT at 6-8 weeks had persistent measurable calcitonin at follow up (18). Brauckhoff and cols. reported that in 12 patients destined to achieve an undetectable post-operative calcitonin level, the CT was undetectable by 24 hours in 8 patients, and by 2 weeks in an additional 4 patients (14). Our data is consistent with the studies reported above and suggests that the one-month CT value is a valuable tool to risk stratify patients for future follow up.

As with any retrospective study, there are important limitations that need to be considered. Because calcitonin and CEA levels were routinely done at set intervals (1 month, 2-3 months, 6 months, and 12 months post-operative), we cannot determine the precise time course of calcitonin and CEA values during the first few weeks after surgery. Secondly, while these patients have demonstrated an excellent response to therapy at the 1 year time point and appear to be cured, previous studies have suggested that disease recurrence can be seen in 5-15% of patients with longer follow up (2). Finally, the definition of undetectable calcitonin varied between patients as the assays used in individual patients varied between institutions and over time. It is recognized that in very rare cases, there is serum calcitonin negative MTC pre-operative (19). However, we don't believe that this changes this results, since none of the patients that had undetectable calcitonin turned out to have structural evidence of disease.

In conclusion, measurements of serum calcitonin values as early as 1 month after surgery can provide meaningful insights into the likelihood that a curative surgical procedure has been performed. It is known that patients that reaches excellent response to therapy, the incidence of recurrence are low (2), independently of initial anatomic staging. In this context, this study shows that rather than waiting 3-6 months to find out if surgery was effective, this early response to therapy assessment can be used to create realistic expectations about their disease status and prognosis shortly after definitive surgery.
